# A Facile and Sensitive Method for Quantification of Cyclic Nucleotide Monophosphates in Mammalian Organs: Basal Levels of Eight cNMPs and Identification of 2',3'-cIMP

**DOI:** 10.3390/biom4041070

**Published:** 2014-12-12

**Authors:** Xin Jia, Benjamin M. Fontaine, Fred Strobel, Emily E. Weinert

**Affiliations:** Department of Chemistry, Emory University, Atlanta, GA 30322, USA; E-Mails: xin.jia@emory.edu (X.J.); bfontai@emory.edu (B.M.F.); fstrobe@emory.edu (F.S.)

**Keywords:** liquid chromatography, tandem mass spectrometry, LC-MS/MS, 3',5'-cyclic nucleotides monophosphate, 2',3'-cyclic nucleotides monophosphate, inosine 2',3'-cyclic monophosphate, mammalian cNMP basal levels

## Abstract

A sensitive, versatile and economical method to extract and quantify cyclic nucleotide monophosphates (cNMPs) using LC-MS/MS, including both 3',5'-cNMPs and 2',3'-cNMPs, in mammalian tissues and cellular systems has been developed. Problems, such as matrix effects from complex biological samples, are addressed and have been optimized. This protocol allows for comparison of multiple cNMPs in the same system and was used to examine the relationship between tissue levels of cNMPs in a panel of rat organs. In addition, the study reports the first identification and quantification of 2',3'-cIMP. The developed method will allow for quantification of cNMPs levels in cells and tissues with varying disease states, which will provide insight into the role(s) and interplay of cNMP signalling pathways.

## 1. Introduction

Cyclic nucleotides adenosine 3',5'-cyclic monophosphate (3',5'-cAMP) and guanosine 3',5'-cyclic monophosphate (3',5'-cGMP) are essential mammalian metabolites that function primarily within the cell as secondary messengers. 3',5'-cAMP and 3',5'-cGMP have been investigated for decades and have been found to perform crucial roles in regulating cellular metabolism and mediating actions of numerous mammalian hormones and neurotransmitters [1,2]. The existence of additional cyclic nucleotides, including cytidine 3',5'-cyclic monophosphate (3',5'-cCMP), inosine 3',5'-cyclic monophosphate (3',5'-cIMP) and 2',3'-cyclic nucleotide monophosphates (2',3'-cNMPs), in mammalian tissues and cell lines has previously been reported [3,4,5]. However, while their potential roles in tumorigenesis, cellular signal transduction and post-injury mechanisms have been suggested, their signalling pathways have yet to be elucidated [6,7,8].

Of the atypical cyclic nucleotides, 3',5'-cCMP has been the best studied, having previously been found in pmol/g concentration ranges in various mammalian tissues [4]. Furthermore, 3',5'-cCMP was found to occur at increased concentrations in the brain and in dividing tissues (such as regenerating livers), while putative proteins involved in cCMP signalling pathways were also reported 20–30 years ago, but have yet to be identified and fully characterized [9,10,11,12,13]. In addition to cCMP, 3',5'-cIMP has been identified as an endogenous product released from a variety of rat organs using fast atom bombardment mass spectrometry [5]. Recently, 3',5'-cIMP has been shown to be synthesized by purified soluble guanylyl cyclase (sGC) and by sGC in porcine coronary arteries [14,15]. Furthermore, CadD, an enzyme found in the pathogenic bacterium *Leptospira interrogans*, has been recently reported as a cAMP specific deaminase, resulting in the production of 3',5'-cIMP [7]. This conversion has suggested the possibility that cells also sense 3',5'-cIMP levels [7,16].

In addition to 3',5'-cNMPs, recent reports have detailed the identification and putative roles of some 2',3'-regioisomers. 2',3'-cAMP was first detected in perfused rat kidney using liquid chromatography coupled with tandem mass spectrometry (LC-MS/MS) [17]. Additional studies have suggested that tissue injury triggers mRNA degradation, leading to production of 2',3'-cAMP that is converted through the action of the enzyme 2',3'-cyclic nucleotide phosphodiesterase to endogenous adenosine, which upon binding to various adenosine receptors can protect against acute organ injury, as well as have profound effects on the cardiovascular system [8,18,19,20,21,22,23,24]. Recently, 2',3'-cAMP and cGMP have been shown to correlate with leaf wounding stress in *Arabidopsis*, further suggesting that these nucleotides may be important in post-injury mechanisms [25]. 2',3'-cCMP and 2',3'-cGMP also were reported in two mammalian cell lines (HEK293T and HuT-78), however their role is unknown [3]. Such observations imply that multiple 2',3'-cNMPs may be involved in various cellular pathways, including mechanisms to protect against tissue injury.

Despite the progress that has been made to establish the role of cNMPs in signalling pathways, quantitation of mammalian tissue distributions has been challenging. Previous studies have reported problems with identifying cNMPs due to interference from tissue components and lack of an efficient extraction protocol for analyzing extremely low concentrations of cNMPs in mammalian organs [26,27]. Since the 1970s, various procedures for measurement of extracted cNMPs in organs have required initial separation by thin layer chromatography (t. l. c.) or ion exchange resin (e.g., dowex resin) [26,28,29,30]. However, those protocols were time-consuming and resulted in low sensitivity, which made rapid analysis of large numbers of tissue samples that contain low concentrations of cNMPs problematic. Radioimmunoassay techniques have also been used due to the improved sensitivity and simplified quantification protocol, but the use of radioactive isotopes can introduce difficulties [31,32]. To date, most reports measure relative levels of either cAMP or cGMP following an external stimulus using commercially available enzyme-linked immunosorbent assays (ELISAs), rather than absolute concentrations [33,34,35]. Using ELISAs has significant advantages, including safety of handling, minimal training requirements and ease of waste disposal. Unfortunately, despite the adequate sensitivity and cost, cross-reactivity may occur with the secondary antibody, resulting in over-estimation of the analyte-of-interest [36]. Therefore, a sensitive and high-throughput method to analyze low concentrations of multiple cNMPs in tissues should find utility in both research and clinical laboratories.

Use of LC-MS/MS techniques provides the advantages of high sensitivity, high-throughput data analysis, and widespread instrumentation [37,38,39,40,41]. It has quickly been adapted as a main method for quantitative metabolomics and nucleotideomics in the past decade [37,38,39]. Furthermore, LC-MS/MS has found significant utility in studying bio-markers in clinical laboratories [42,43]. However, current LC-MS/MS protocols for extraction of cNMPs from mammalian tissues have typically quantified only one to two cNMPs, rather than monitoring a wide range of cNMP levels [44,45,46]. Previous work has observed the negative impact of matrix effects in organ extraction samples analyzed by LC-MS/MS, specifically phospholipids in biological extracts that can be ionized competitively with the analytes-of-interest, decreasing the sensitivity and reproducibility of the method [47,48,49,50]. However, in most published organ extraction protocols, a solution to solve such matrix effects has not been offered [51,52].

Since previous studies suggest that both 3',5'- and 2',3'-cNMPs may be biologically important, an efficient and sensitive protocol has been developed and validated for extraction and quantification of multiple cNMPs simultaneously in various systems (organs, cell lines, *etc.*) using instrumentation available in the majority of research institutions. By utilizing standard instrumentation and a readily available internal standard (IS), this procedure should be useful for a wide variety of researchers and will facilitate further investigations into the physiological roles of cNMPs.

## 2. Results and Discussion

### 2.1. Optimization of LC-MS/MS Analytical Method

**Figure 1:**
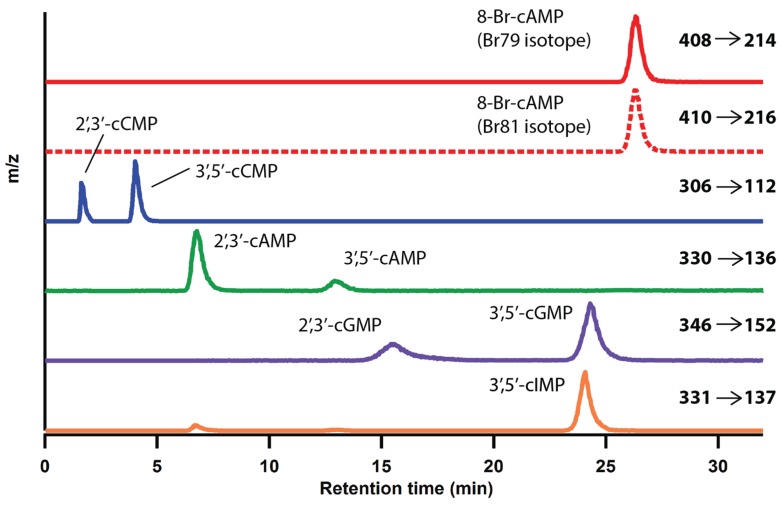
Reconstructed ion chromatogram of authentic cNMPs using Xcalibur software. From top to bottom, transition 408 → 214 *m/z* was monitored and reconstructed for ^79^Br-cAMP (Top, red trace) and transition 410 → 216 *m/z* was monitored and reconstructed for ^81^Br-cAMP (dotted red trace); transition 306 → 112 m/z was monitored and reconstructed for 2',3'-cCMP and 3',5'-cCMP (blue trace); transition 330 → 136 *m/z* was monitored and reconstructed for 2',3'-AMP and 3',5'-cAMP (green trace); transition 346 → 152 *m/z* was monitored and reconstructed for 2',3'- cGMP and 3',5'-cGMP (purple trace); transition 331 → 137 *m/z* was monitored and reconstructed for 3',5'-cIMP (orange trace).

Development of the current method to extract and quantify cNMPs from mammalian tissues began with optimization of the separation and detection protocols by LC-MS/MS. The LTQ mass spectrometer in a Thermo Electron LTQ-FTMS system was used for all analyses, as similar systems are typically available to researchers through departmental or university instrument cores due to their popularity because of their ease of use and utility in protein studies. Many existing protocols studying metabolomics use a combination of ultra-performance liquid chromatography (UPLC) coupled with a triple quadrupole MS for greater sensitivity and precision [45,53,54]. However, UPLCs often are not readily available to non-specialist investigators. Initial work focused on optimizing the separation of various cNMP isomers so that unique retention times can be used as identifiers for each cNMP. Different types of LC columns such as HILIC, fluoro phenyl, and C-18 were tested due to their abilities to separate polar compounds and acidic compounds that contain aromatic substituents [55]. Following testing of various columns, a reverse phase C-18 column was chosen due to its stability, as well as reproducibility and peak shapes when separating seven cNMPs. Positive electrospray ionization (ESI) was used to fragment cNMPs in the mass spectrometer using a 0.1% formic acid buffer to enhance protonation of the cNMP species. Positive ion MS/MS in the LTQ yielded much greater sensitivity compared to other ion modes tested and produced multiple fragments that could be used simultaneously to unambiguously identify analytes, differentiating the 2',3'- and 3',5'-regioisomers (Supplemental Table S1 and Supplemental Figure S1). Chromatographic separation of all seven cNMPs and the IS (8-Br-cAMP) was achieved by liquid chromatography, prior to using the mass spectrometer to distinguish analytes based on mass to charge ratio. Figure 1 depicts the reconstructed ion chromatograms of the different protonated cNMPs. The 2',3'- and 3',5'-isomers of each cNMP are visualized by fragment ions correspond to protonated base, which were used for peak integration. Previously, due to the structural similarity of purine and pyrimidine cyclic nucleotides, clear separation by LC was difficult achieve [56]. Optimization of the LC-MS/MS system to fully separate all cNMPs also can be difficult to accomplish, as has been observed in a number of publications that utilize LC-MS/MS systems [3,51,52]. As shown in Table 1, although cGMP and cIMP are structurally similar, the current method can readily separate the two analogues due to the difference in protonated precursor ion [M + H]^+^ mass to charge ratio (15 *m/z*). The improved peak shapes on the reconstructed ion chromatogram (Figure 1) yielded separation of cGMP and cIMP (RTs of cGMP and cIMP are 24.3 and 24.1 min, respectively, Table 1), allowing for accurate identification.

8-Bromoadenosine 3',5'-cyclic monophosphate (8-Br-cAMP) was employed as an internal standard (IS) in the present method to quantify the extracted cNMPs. 8-Br-cAMP was chosen because it is readily commercially available, relatively inexpensive, and not naturally present in mammalian cells. In addition, the cyclic phosphate group of 8-Br-cAMP is shared with the cNMPs, resulting in similar ionization efficiencies. Moreover, the two naturally occurring isotopes of bromine allow for unequivocal identification and quantification of 8-Br-cAMP in extracted samples.

**Table 1:** Mass spectrometric parameters for the measured transitions of cyclic nucleotide monophosphates (cNMPs) and internal standard (8-Br-cAMP; IS).

Parameters	2',3'-cAMP	3',5'-cAMP	2',3'-cCMP	3',5'-cCMP	2',3'-cGMP	3',5'-cGMP	3',5'-cIMP	8-^79^Br-cAMP	8-^81^Br-cAMP
**[M + H]^+^ (*m/z*)**	330.1	330.1	306.1	306.1	346.1	346.1	331.0	408.0	410.0
**Production (*m/z*)**	136.1	136.1	112.1	112.1	152.1	152.1	137.1	214.0	216.0
**Ret. time (min)**	6.8	12.9	1.6	4.0	15.6	24.3	24.1	26.3	26.3
**LOD (fmol)**	273	153	171	455	94	487	219	NA	NA
**LOQ (fmol)**	910	510	570	1517	313	1623	730	NA	NA

[M + H]^+^ = protonated molecular mass; NA = not applicable; LOD = limit of detection; LOQ = limit of quantitation.

### 2.2. LC-MS/MS Method Calibration and Limits of Detection

Limits of detection (LOD) using the developed LC-MS/MS protocol for 2',3'-cAMP, 3',5'-cAMP, 2',3'-cCMP, 3',5'-cCMP, 2',3'-cGMP, 3',5'-cGMP and 3',5'-cIMP were calculated [57,58] as 273 fmol, 153 fmol, 171 fmol, 455 fmol, 94 fmol, 487 fmol and 219 fmol, respectively (Table 1). For all samples, in order to minimize intra-assay variability, each sample was measured in 2–3 separate runs. The average and standard deviation (or range) were calculated and reported for each sample. A set of standards to generate calibration curves was included at the beginning of each LC sequence to account for any day-to-day variability in ionization efficiency or retention times of cNMPs.

### 2.3. Optimization of Extraction Method

Difficulty with organ extractions has previously been reported due to low levels of cNMPs and interference from the complex organ matrix due to inefficient extraction protocols [26,47,48,49,50]. In addition, published protocols typically have focused on identifying or quantifying only a small number of cNMPs in cells and often are not adapted to analyze organ extracts [3,40,45]. The lack of an efficient extraction and analysis protocol to study multiple cNMPs has made establishing tissue distributions of all cNMPs very challenging. As cNMPs are of interest as both established and putative signalling molecules, it is crucial to develop a versatile extraction protocol using standard instrumentations that many researchers can employ in order to establish the role of cNMPs in mammalian tissues, as well as in other systems.

**Figure 2:**
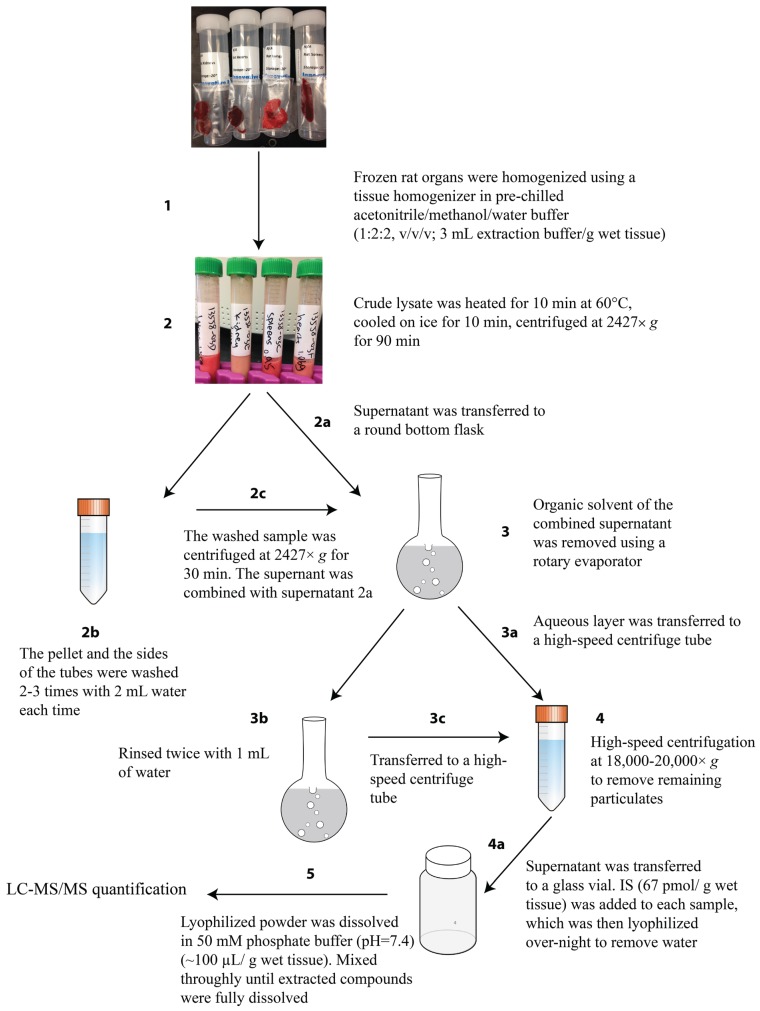
Workflow chart of cNMPs extraction from rat organs. Brain, spleen and heart samples require high-speed centrifugation (step 4) at 18,000× *g*. Liver, lung and kidney samples require high-speed centrifugation (step 4) at 20,000× *g*.

An outline of the optimized extraction protocol is depicted in Figure 2. In order to achieve the best results, a series of optimization experiments were performed to maximize internal standard (IS) recovery and cNMP signal intensities in LC-MS/MS. Frozen rat organs were homogenized in pre-chilled extraction buffer containing the phosphodiesterase (PDE) inhibitors EDTA and theophylline (step 1). Different combinations and ratios of organic and aqueous solvent were evaluated and the reported extraction mixture (acetonitrile/methanol/water 1:2:2, v/v/v) yielded the best analyte recovery. A solubility test was conducted to ensure extracted cNMPs were dissolved in the extraction mixture. Solubilities of the IS and seven cNMPs are greater than or equal to 1 mM in extraction mixture. As a previous publication indicates, cAMP and cGMP levels in mammalian cells are well below the micromolar range, the chosen extraction mixture will therefore dissolve all extracted cNMPs [4,9,45].

Following homogenization, the crude lysate was heated (Figure 2, step 2) to denature enzymes that could bind, synthesize, or hydrolyze cNMPs, thereby decreasing the possibility of altered basal levels. Thermal stability of the seven cNMPs and IS were tested under the heat denaturation conditions and all were found to be thermally stable. cNMP concentrations were 87%–141% compared to the reference samples, indicating that heating does not degrade cNMPs (Supplemental Table S2). Moreover, a stability test of cAMP in water exposed to the extraction conditions did not result in formation of cIMP, suggesting that 2',3'-cIMP and 3',5'-cIMP detected in rat organs were not degradation products of 3',5-cAMP and 2',3'-cAMP. A similar test was also conducted in crude organ lysate and addition of 3',5'-cAMP to an organ extract did not cause an increase in 3',5'-cIMP levels, indicating that 3',5'-cIMP, and likely 2',3'-cIMP, detected in organ samples were not deaminated products of 3',5'- and 2',3'-cAMP (Supplemental Table S3) [59]. Low-speed centrifugation following step 2 was used to remove lipids, cell walls and denatured proteins from extracted small molecules in the supernatant. Results have shown that low centrifugation at ~3000× *g* is sufficient to remove most insoluble material, as the supernatant (Figure 2, step 3) generated after treatment appears to be clear, except for liver samples, which are particularly complex. Homogenization, heating and centrifugation were performed in the same conical tube to minimize loss of extracted cNMPs.

**Figure 3:**
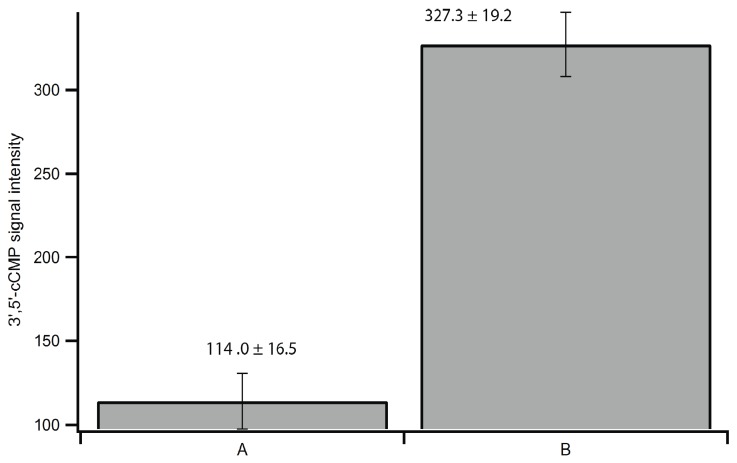
LC-MS/MS signal intensity of 3',5'-cCMP in rinsing tests. Crude lysate from a brain extract was split into two portions A and B, heat extractions were performed independently. (**A**) Sample was treated without extensive rinsing of tube walls; (**B**) sample was treated with extensive rinsing of tube walls (Figure 2, step 2b).

Rinsing of the sides of the tube walls and pellet during step 2b was performed to ensure transfer of any residual cNMPs on the tube walls or pellet. Figure 3 shows that 3',5'-cCMP recovery improved by over three fold when tube walls were washed extensively, suggesting that cNMPs can stick to the tube walls or to fine particulate matter removed during centrifugation. 8-Br-cAMP is less soluble than most natural cNMPs, and all cNMPs tend to stick to the tube walls and/or particles within the tubes, making the rinsing steps (Figure 2, steps 2b and 3b) crucial for complete recovery of extracted cNMPs and IS.

The combined supernatants from steps 2a–2c were concentrated using a rotary evaporator to remove organic solvent, as the organic solvents often resulted in melting of the mixture on the freeze-dryer. Depending on the lyophilizer/free-dryer used, this step can be eliminated and the supernatant can be concentrated without use of the rotary evaporator. As discussed above, an extra rinse (Figure 2, step 3b) was included when transferring the aqueous layer to a high-speed centrifuge tube (Figure 2, step 3a–3c) to ensure complete recovery of extracted cNMPs.

**Figure 4:**
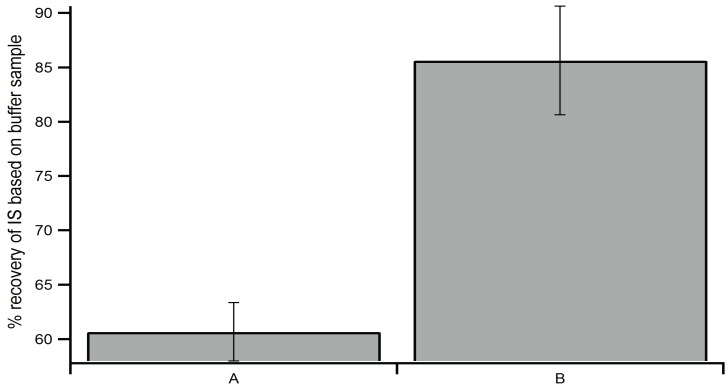
Percent recovery of IS signal in samples without and with high-speed centrifugation. Heat extractions were performed independently. (**A**) Sample was treated without high-speed centrifugation step; (**B**) sample was treated with the high-speed centrifugation step (Figure 2, step 4).

The high-speed centrifugation step was employed to remove remaining particulate material, thereby improving separation on the LC column and sensitivity of the LC-MS/MS. Results in Figure 4 show that IS recovery was improved to ~90% by utilizing a high-speed centrifugation step in sample preparation. As previously mentioned, steps to eliminate matrix effects by removing cellular debris were crucial for improving sensitivity. High-speed centrifugation eliminated the remaining particulates in the sample, which resulted in improved sensitivity. Filtering samples using a number of different brands of syringe filters to remove remaining matrix components also was tested; however, including a filtering step did not decrease the prevalence of sample components that limited sensitivity and clogged the column. Additionally, the signal intensity of the IS on LC-MS/MS is crucial in quantifying putative cNMP levels, since the IS signal and concentration of the cNMP analytes are inversely proportional, low IS signal will lead to over-estimation of cNMP concentration. Treating brain, spleen and heart samples with high-speed centrifugation at 18,000× *g* is sufficient to achieve the reported percent recovery. However, due to the nature of the organs, centrifugation at 20,000× *g* or higher is required for liver, lung and kidney samples. Finally, the clear supernatant extracted from each organ was transferred to a glass vial, followed by addition of the IS and lyophilization over-night to remove water (Figure 2, step 4a). The lyophilized powder containing extracted cNMPs was re-dissolved in 50 mM phosphate buffer (pH = 7.4) (~10 µL/g wet tissue) before analysis by LC-MS/MS.

### 2.4. Method Validation and Reproducibility Tests

**Figure 5:**
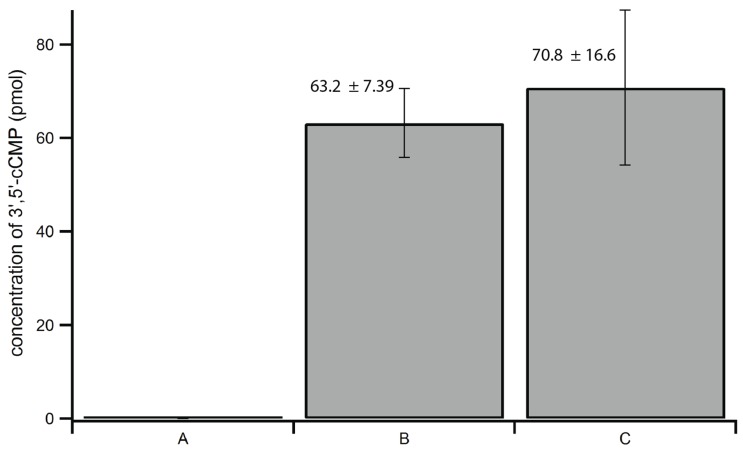
Method validation test shows recovered 3',5'-cCMP (added 60 pmol 3',5'-cCMP) following addition at various time points during the extraction protocol. A brain crude lysate was split into three portions A, B and C, and heat extractions were performed independently (refer to Experimental section 3.9). (**A**) Basal level of 3',5'-cCMP; (**B**) 60 pmol of 3',5'-cCMP was added with the crude lysate (Figure 2, step 1); (**C**) 60 pmol of 3',5'-cCMP was added with IS (Figure 2, step 4a). Data in tabulated form can be found in Supplemental Table S5.

In order to validate the present extraction protocol [60], extraction replicates using a frozen brain were performed. Sample A in Figure 5 shows the basal level of 3',5'-cCMP detected in the brain sample, which was below the LOD. To samples B and C, 60 pmol of 3',5'-cCMP were added at various points during extraction protocol to determine if the developed method is able to isolate all of the cNMPs within biological samples. Addition of 60 pmol 3',5'-cCMP to the crude lysate (Figure 2, step 1) or with IS (Figure 2, step 4a) resulted in 105% ± 12% and 118% ± 28% recovery of the added 3',5'-cCMP, respectively. These results demonstrate that the present extraction protocol is efficient in recovering extracted cNMPs. Also, addition of IS at various points during the extraction protocol does not affect the outcome of the extraction procedures (Supplemental Table S4).

To test the reproducibility of the protocol, the optimized method was performed using rat brains. A frozen brain was split into three portions, which were extracted and quantified independently (refer to Experimental section 3.10). As shown in Table 2, the relative standard deviation of each calculated cNMP concentration ranges from 2.1%–20.0%. It is worth noting that the relative standard deviation may have suffered, particularly for 3',5'-cCMP, due to the extremely low concentration in each organ portion. The relative standard deviations for the calculated concentrations that are several fold above the LOD (2',3'-cAMP, 3',5'-cAMP and 3',5'-cGMP) are less than or equal to 10% (Table 2).

**Table 2:** Reproducibility of the method. A rat brain was split into three portions, and cNMPs were extracted and quantified independently. Results are reported in pmol/g of tissue.

Sample	2',3'-cAMP	3',5'-cAMP	2',3'-cCMP	3',5'-cCMP	2',3'-cGMP	3',5'-cGMP	3',5'-cIMP
**Portion 1**	9.9 ± 0.2	88.7 ± 12.3	1.2 ± 0.9	0.9 ± 0.7	2.5 ± 0.01	10.1 ± 2.3	2.6 ± 0.9
**Portion 2**	9.6 ± 0.3	92.3 ± 2.9	ND	0.6 ± 0.4	1.8	8.7 ± 0.3	2.3 ± 0.3
**Portion 3**	9.7 ± 0.2	87.9 ± 4.8	1.3	0.6 ± 0.4	1.8 ± 0.5	8.3 ± 0.7	3.1 ± 0.3
**Average**	9.7 ± 0.2	89.6 ± 2.3	1.3 ± 0.1	0.7 ± 0.1	2.0 ± 0.4	9.0 ± 0.9	2.7 ± 0.4
**Inter-run Precision**	2.1	2.6	7.7	14.3	20.0	10.0	14.8

N/D—Not detected, concentration below LOD; Precision—relative standard deviation in %.

### 2.5. Applications

**Figure 6:**
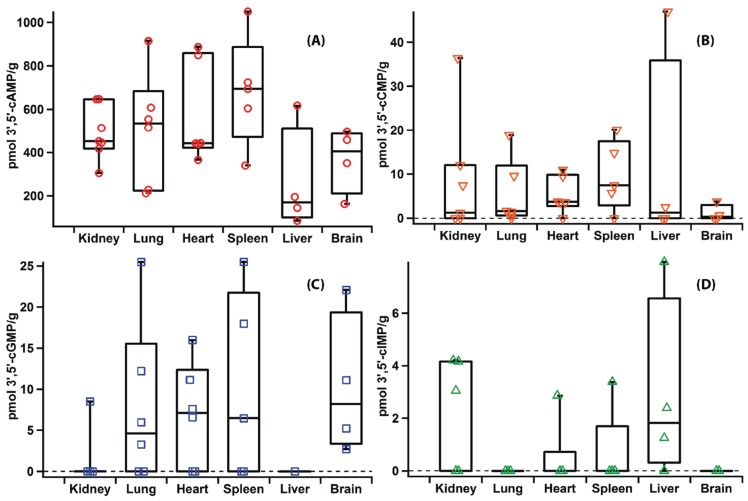
Levels of extracted 3',5'-cNMPs (pmol/g wet tissue) in rat organs. Each point represents the level measured in a replicate from a different rat. (**A**) 3',5'-cAMP (**B**) 3',5'-cCMP (**C**) 3',5'-cGMP (**D**) 3',5'-cIMP. Whisker top: 90 percentile, box top: 75 percentile, box middle: 50 percentile, box bottom: 25 percentile and whisker bottom: 10 percentile.

**Figure 7:**
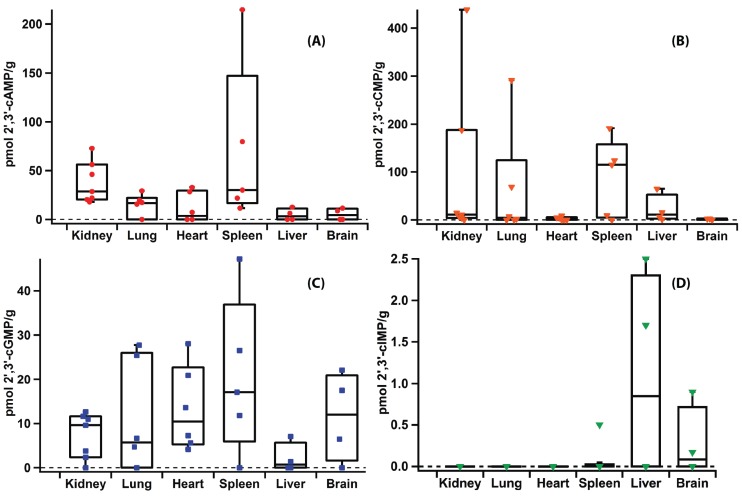
Levels of extracted 2',3'-cNMPs (pmol/g wet tissue) in rat organs. Each point represents the level measured in a replicate from a different rat. (**A**) 2',3'-cAMP; (**B**) 2',3'-cCMP; (**C**) 2',3'-cGMP; (**D**) 2',3'-cIMP. Whisker top: 90 percentile, box top: 75 percentile, box middle: 50 percentile, box bottom: 25 percentile and whisker bottom: 10 percentile.

The existence of additional cyclic nucleotides beyond the paradigmatic second messengers 3',5'-cAMP and cGMP has been reported in the literature for a number of years [3,5,8,61]. Therefore, the optimized extraction and quantification protocol was used to detect levels of previously identified, as well as novel, cyclic nucleotides in mammalian organs. Using t. l. c., previous work has found that the concentration of mammalian 3',5'-cGMP is several fold lower than that of 3',5'-cAMP in mammalian organs (488 pmol/g extracted 3',5'-cAMP and 119 pmol/g extracted 3',5'-cGMP in rat heart; 11.8 pg/mg extracted 3',5'-cAMP and 5.2 pg/mg extracted 3',5'-cGMP in rabbit pancreas; all measurements listed as per gram of organ weight) [26,45]. The concentration of 3',5'-cCMP in rat organs also has been previously determined using radioimmunoassay [4] and 3',5'-cIMP and 2',3'-cAMP have been detected in various rat organs [5,17]. Therefore, a panel of rat organs, including brain, spleen, heart, kidney, lung and kidney, was chosen to validate previous studies and to establish tissue distribution of cNMPs in major rat organs. As shown in Figure 6 and Figure 7, eight cNMPs were detected and quantified in the majority of rat organs studied. The ability to analyze eight cNMPs simultaneously in a single run demonstrates the efficiency of the developed method. The concentrations of 3',5'-cGMP, 3',5'-cAMP and 3',5'-cCMP (Figure 6, Supplemental Table S6) agree with the previously reported values within less than an order of magnitude [4,26,45]. In addition, the range of cNMP levels detected in organs from different rats confirms the biological variability between animals that has previously been reported.

In addition to 3',5'-cAMP and cGMP, 2',3'-cAMP has previously been detected in rat kidney and neurons, while 2',3'-cGMP has been detected and quantified in cellular systems [3,17]. This report details the first measurements of 2',3'-cGMP in rat tissues, which should aid in the study of its putative role in post-injury mechanisms (Figure 7, Supplemental Figures S1 and S2). In addition, 3',5'-cIMP has been previously reported as an endogenous product found in a variety of rat organs using t. l. c. and fast atom bombardment mass spectrometry [5]. The present study confirms that 3',5'-cIMP is an endogenous molecule produced in the heart, kidney, spleen and liver (Figure 6, Supplemental Table S6). As a recent publication has suggested a possible role of 3',5'-cIMP in cellular signal transduction, quantification of 3',5'-cIMP in healthy tissues will be useful in establishing relevant physiological concentrations [7,16]. 2',3'-cIMP also was detected in a number of the rat organs studied, although its role *in vivo* has yet to be proposed (Figure 7, Supplemental Table S3). We believe the present extraction protocol will help identify and quantify 3',5'- and 2',3'-cIMP levels in various disease states and promote further study of their potential roles as signalling molecules.

It is worth mentioning that the concentrations of 2',3'-cCMP, 3',5'-cCMP, 2',3'-cIMP and 3',5'-cIMP in brain, heart and liver samples are close to the LOD of the method and therefore are associated with relatively high standard deviations (Figure 6 and Figure 7, Supplemental Table S6). While the higher deviations potentially could be due to variability in the method, the validation experiment described above suggests that bio-variability between animals and organs plays a larger role than variability due to the method. For low abundance cNMPs, the precision of calculated concentrations can be improved by increasing the injection volume or concentration of samples. However, caution should be used when attempting to produce extremely concentrated samples as cNMPs can be lost if the entire extract is not completely dissolved. In addition, analysis of samples using a triple quadrupole MS instrument would likely improve sensitivity and the described method can be readily adapted if researchers have access to such an instrument. Improving sensitivity through either method will be important for future studies analyzing small organs such as rat spleen and heart (~0.7 g and ~0.8 g, respectively), as the total amounts of cNMPs in smaller organs are close to the LOD.

**Figure 8:**
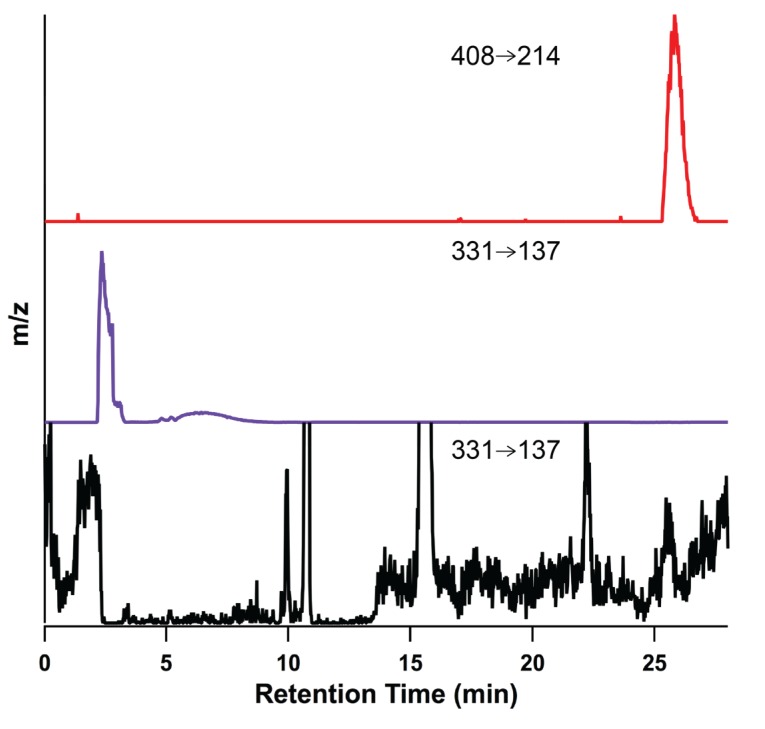
Reconstructed ion chromatogram of IS (top, red trace), authentic 2',3'-cIMP (purple trace), and extracted 2',3'-cIMP (bottom, black trace) from male rat brain using Xcalibur software. Transition 331 → 137 *m/z* was monitored and reconstructed for extracted 2',3'-cIMP.

During the analysis of cNMP tissue distributions, a peak proposed to correspond to 2',3'-cIMP was identified. An authentic standard of 2',3'-cIMP was synthesized [62,63,64] and used to verify the identity of the extracted compound based on molecular weight, fragmentation pattern, and retention time. As can be seen in Figure 8, both authentic and extracted 2',3'-cIMP elute at 2.4 min, supporting our identification of 2',3'-cIMP in mammalian organs (Figure 8). The other peaks in the extracted 2',3'-cIMP chromatogram include 3',5'-cIMP and 3',5'-cAMP that has incorporated a single ^13^C, which results in the same low resolution mass as cIMP.

**Figure 9:**
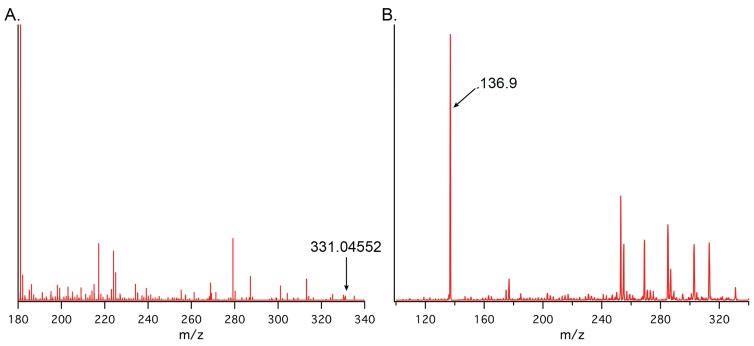
High resolution MS (**A**) and MS/MS (**B**) spectra of 2',3'-cIMP extracted from rat brain. Authentic 2',3'-cIMP: 331.04525; extracted: 331.04552. Authentic 2',3'-cIMP [BH_2_]^+^: 136.9; extracted: 136.9.

Additionally, the high resolution spectrum of extracted 2',3'-cIMP yielded *m/z* of 331.0455 (authentic 2',3'-cIMP = 331.04525) and, based on the fragmentation of authentic 2',3'-cIMP (Supplementary Figure S4) and on previously published fragmentation patterns of 2',3'-cGMP [65], the predicted product ion corresponding to the protonated inosine base [BH_2_]^+^ was observed at *m/z* 136.9 (authentic 2',3'-cIMP[BH_2_]^+^ = 136.9, Figure 9). 2',3'-cIMP was detected, although often below the limit of quantitation, in extracts from brain, kidney, spleen, heart, and liver (Supplemental Table S6). Therefore, we believe that we have detected 2',3'-cIMP in a panel of mammalian organs. Based on the precedence of enzymatic production of 3',5'-cIMP from 3',5'-cAMP, 2',3'-cIMP also may be formed enzymatically *in vivo* through the deamination of 2',3'-cAMP [7]. If so, deamination of 2',3'-cAMP may play a role in modulating levels of 2',3'-cAMP to alter the downstream effects of the mammalian 2',3'-cAMP-adenosine pathway [8]. To our knowledge, this study presents the first detection of 2',3'-cIMP and the first quantification of 3',5'-cIMP in a panel of mammalian organs, laying the groundwork for future studies of the biochemistry and physiology of both cIMP isomers.

### 2.6. Versatility of the Method

To further extend the method, its utility was tested on mammalian cells. NIH-3T3 cells were studied using the developed LC-MS/MS protocol. As shown in Table 3, most cNMPs, except 3',5'-cCMP, 2',3'-cIMP and 3',5'-cIMP, were detected and quantified in this particular cell line. Extracted samples from cell lines were much cleaner and required fewer washing and transfer steps (due to the decreased volume) compared to organ extractions and the data showed improved sensitivity and reproducibility. On-going experiments have found that the present method also can be used to quantify levels of cNMPs in *Escherichia coli* samples.

**Table 3:** Measured concentrations of eight cNMPs in NIH-3T3 cell line reported as mean ± SD (pmol/10^6^ cells). Each cNMP concentration was calculated from three separated samples (1, 2 and 3), and each sample was analyzed in two separate runs.

Replicate	2',3'-cAMP	3',5'-cAMP	2',3'-cCMP	3',5'-cCMP	2',3'-cGMP	3',5'-cGMP	2',3'-cIMP	3',5'-cIMP
**1**	5.1 ± 0.3	4.8 ± 0.1	1.3 ± 0.1	N/D	2.1 ± 0.1	N/D	N/D	N/D
**2**	N/D	167.1 ± 35.0	N/D	N/D	32.0 ± 2.3	17.2 ± 0.3	N/D	N/D
**3**	N/D	99.8 ± 0.2	1.8	N/D	19.9 ± 0.2	8.0 ± 3.7	N/D	N/D
**Average**	5.1	90.6 ± 81.6	1.6 ± 0.3	N/D	18.0 ± 15.0	12.6 ± 4.6	N/D	N/D

N/D—not detected, concentration below LOD.

### 2.7. Discussion

The aim of this work was to develop an efficient and versatile LC-MS/MS protocol that can detect and quantify multiple cNMPs concurrently in biological and cellular samples using standard instrumentation. There are several key steps that were discovered in developing the current protocol. First, cNMPs tend to stick to the centrifuge tube walls during extraction, regardless of whether glass or plastic tubes are used; therefore, an extensive rinsing step is key to ensuring complete recovery of extracted cNMPs. Secondly, matrix effects resulted in unfavourable competitive ionization with the analytes-of-interest, which is common in complex biological samples. However, introduction of a high-speed or ultra-centrifugation step to remove remaining particulate can greatly reduce the matrix effect and increase sensitivity. Despite high-speed centrifugation, liver samples were found to clog the ion transfer tube on the LC-MS/MS; cleaning the tube after every 4–5 samples also can help to maintain high sensitivity during high throughput sample analysis. In addition, large numbers of sample runs (~50–70) lead to the build-up of extracted compounds from organs on the column. This problem is easily solved by washing with organic solvents (e.g., acetonitrile or DMF) or switching buffer systems (e.g., triethanolamine HCl, pH 5.5) to remove the remaining material. Lastly, while the results of the current work were achieved using a quadrupole ion trap, this method could easily be adapted to a triple quadrupole detector, which would further increase the sensitivity of the current method.

One advantage of the present extraction protocol is that unlike quantification protocols using isotopically labelled cNMPs or expensive/synthetically challenging nucleotide analogues as the internal standard, affordable and commercially available 8-Br-cAMP was employed as an IS to allow for quantification of cNMPs and to account for any losses during sample preparation. 8-Br-cAMP was chosen because it is not naturally present in mammalian cells, is readily commercially available, and is relatively inexpensive (particularly in comparison to previously described internal standards) [3,45]. Due to the structural similarity between the internal standard and the cNMP analytes, the ionization efficiency of 8-Br-cAMP is similar to the natural cNMPs.

The optimized extraction and quantification protocol was used to detect levels of previously identified, as well as novel cyclic nucleotides in mammalian organs. The quantification of additional atypical nucleotides should aid in identifying their roles *in vivo*. 2',3'-cAMP and cGMP have been shown to correlate with stress in perfused kidney and in *Arabidopsis* following leaf wounding, suggesting that these nucleotides may be important in post-injury mechanism [25]. Furthermore, the potential roles of 3',5'-cCMP, cIMP, cUMP, cTMP and cXMP as secondary messengers have been proposed for decades and are still under investigation [3,5,27,61]. Previous studies have revealed the broad substrate specificity of purified soluble guanylyl cyclase (sGC) and its ability to synthetize a variety of atypical cNMPs at various rates; however, little is known about their physiological and biochemical roles in mammalian systems [15,66,67]. The developed protocol allows for the detection and quantification of a large panel of cNMPs, which will provide a powerful tool in analyzing cNMPs levels in mammalian systems.

## 3. Experimental

### 3.1. Materials

Organs from Sprague-Dawley rats (brain, spleen, lung, kidney, heart and liver) were purchased from Innovative Research Inc. (Novi, MI, USA). According to the supplier, all organs were flash-frozen immediately after harvest and were stored at −80 °C upon receipt. Adenosine 2',3'-cyclic monophosphate (2',3'-cAMP) was purchased from MP Biomedicals (Solon, OH, USA). Adenosine 3',5'-cyclic monophosphate hydrate (3',5'-cAMP) was purchased from TCI America (Portland, OR, USA). Cytidine 2',3'-cyclic monophosphate monosodium salt (2',3'-cCMP) was purchased from Carbosynth (Berkshire, UK). Cytidine 3',5'-cyclic monophosphate (3',5'-cCMP) and guanosine 2',3'-cyclic monophosphate (2',3'-cGMP) were purchased from BioLog (Bremen, Germany). Guanosine 3',5'-cyclic monophosphate sodium salt (3',5'-cGMP), inosine 3',5'-cyclic monophosphate sodium salt (3',5'-cIMP), 8-bromoadenosine 3',5'-cyclic monophosphate (8-Br-cAMP), theophylline and 3-isobutyl-1-methylxathanine (IBMX) were purchased from Sigma-Aldrich (St. Louis, MO, USA). Ethylenediaminetetraacetic acid, disodium salt, dihydrate (EDTA) was purchased from EMD Millipore (Gibbstown, NJ, USA). HPLC-grade acetonitrile and methanol were obtained from Thermo Fisher Scientific (Rockford, IL, USA). Dulbecco’s Modification of Eagle’s Medium (DMEM), Cosmic Calf Serum (CCS), L-glutamine, penicillin G and streptomycin were purchased from Mediatech Inc. (Manassas, VA, USA).

### 3.2. Calibration Curves

Stock solutions of all cNMPs and internal standard 8-Br-cAMP were quantified using a UV-visible spectrophotometer (Cary Series, Agilent Technology, Santa Clara, CA, USA). Calibration curves were constructed by plotting the ratio of peak area for each cNMP/internal standard (IS) against the ratio of concentration of each cNMP/IS in each standard sample. Concentration of IS in each calibration curve is 1 µM, while concentrations of the cNMPs ranged from 0.05–2.5 µM. The concentration range for the calibration curve was chosen because it encompasses the typical concentrations of cNMPs within the experimental samples that are injected on the LC-MS/MS (section 3.6). Calibration curves were generated using a linear regression model.

### 3.3. NIH-3T3 Cell Growth

NIH-3T3 cells were cultured in DMEM supplemented with 10% (v/v) CCS, L-glutamine (2.1 mM), penicillin G (100 IU mL^−1^) and streptomycin (100 μg mL^−1^) were incubated with 5% (v/v) CO_2_ at 37 °C. Cells were passaged at 90%–100% confluency.

### 3.4. cNMP Extraction from Rat Organs

Frozen Sprague-Dawley rat organs (brain, spleen, lung, kidney, heart and liver) were homogenized using a tissue homogenizer (TH, OMNI International, Kennesaw, GA, USA) in pre-chilled acetonitrile/methanol/water buffer (1:2:2, v/v/v; 3 mL extraction buffer/g wet tissue) containing the phosphodiesterase inhibitors EDTA and theophylline (1 mM each) in conical tubes (Celltreat Scientific Products, Shirley, MA, USA). The crude lysate was heated in a water bath for 10 min at 60 °C, cooled on ice for 10 min, centrifuged at 2427× *g* for 90 min and the supernatant transferred to a round bottom flask.

The pellet from the first centrifugation and the sides of the conical tube were washed twice with 2 mL of water each time for brain, spleen, lung, kidney and heart samples and centrifuged at 2427× *g* for 30 min to isolate the supernatant. The supernatant from the wash was combined with the supernatant from the first centrifugation. One additional rinse with 2 mL of water was required for liver due to its size. Combined supernatants containing the extracted cNMPs were transferred to a round bottom flask (Chemglass Life Sciences, Vineland, NJ, USA) and the organic solvent was concentrated using a rotary evaporator (IKA^TM^, RU10 Basic, Wilmington, NC, USA) at 35 °C and the concentrated solution transferred to centrifuge tubes. The round bottom flask was rinsed twice with 1 mL of water each time to ensure complete transfer of cNMPs to the high-speed centrifuge tubes.

High-speed centrifugation of the cNMP solution following rotary evaporation was performed for 30 min at 20,000× *g* for liver and 18,000× *g* for other organs to remove any remaining particulates. The supernatant after high-speed centrifugation was transferred to a glass vial (Wheaton, Millville, NJ, USA) and 67 pmol/g wet tissue of internal standard (IS), 8-Br-cAMP was added. The sample was frozen in liquid N_2_ and lyophilized (Flexi-Dry^TM^, FTS Systems, Warminster, PA, USA) overnight to remove water.

### 3.5. cNMP Extraction from NIH-3T3 Cell Line

The pellet of NIH-3T3 cells was re-suspended in 125 μL pre-chilled extraction buffer (125 µL/10^6^ cells) containing EDTA and theophylline (1 mM each). IS (20 pmol 8-Br-cAMP/ 10^6^ cells) was added to the sample, which was then subjected to 10 cycles of flash-freezing in liquid N_2_, followed by thawing on ice, until the cells appeared lysed by visual inspection. The lysate was heated at 80 °C for 10 min, cooled on ice for 10 min, and centrifuged at 13,000× *g* for 30 min at room temperature to precipitate cellular debris. The supernatant was removed for analysis, and then the pellet and sides of the tube were washed twice with 100 μL of water, centrifuged at 13,000× *g* for 30 min and the supernatants from each centrifugation step were combined. The combined supernatants were lyophilized over-night to remove water.

### 3.6. LC-MS/MS Sample Preparation

Liquid chromatography-tandem mass spectrometry (LC-MS/MS) samples were prepared by re-dissolving lyophilized powder from each extraction in 50 mM phosphate buffer (pH = 7.4) (~100 µL/g wet tissue and 20 µL/10^6^ cells). Re-dissolved samples were thoroughly mixed on a vortex mixer (Analog Vortex Mixer, VWR, Radnor, PA, USA) and then placed in an ultrasonic cleaner (Branson Model 2510, Fisher Scientific, Pittsburgh, PA, USA) for 3 min to fully dissolve all extracted compounds. The vortexing and sonicating steps were repeated two more times to yield clear LC-MS/MS samples.

### 3.7. LC-MS/MS Optimized Conditions

LC-MS/MS experiments were performed using a Thermo Electron LTQ-FTMS system equipped with a Shimadzu autosampler (SIL20AC, Shimadzu, Columbia, MD, USA) and a Dionex Ultimate 3000 dual gradient pump and diode array detector controlled by Xcalibur and DCMSlink software (Thermo Scientific). Samples were separated using a reverse phase (C-18) column (15 mm × 2.1 mm, 2.7 μm, Ascentis Express; guard column (0.5 cm × 2.1 mm, 2.7 μm, Ascentis Express, Sigma Aldrich). 20 µL of sample were analyzed by LC-MS/MS system during each run. Buffer A contained 0.1% formic acid in water and buffer B contained 0.1% formic acid in methanol. The optimized LC protocol (Buffer A/B) for separating cNMPs is as follows: 0–4 min, 0% B; 4–15 min, 0%–1.5% B; 15–20 min, 1.5%–8% B; 20–25 min, 8% B; 25–28 min, 8%–15% B; 28–35 min, 15% B; 35–45 min, 0% B with a flow rate of 0.3 mL/min. After 4 tissue samples, the column was washed using acetonitrile (Buffer C) to ensure removal of all retained cellular compounds. The wash protocol is as follows: 0–2 min, 0%–100% B, 0% C; 2–10 min, 100% B, 0% C; 10–12 min, 100%–0% B, 0%–100% C; 12–20 min, 0% B, 100% C; 20–25 min, 0% B, 100%–0% C; 25–40 min, 0% B, 0% C with a flow rate of 0.3 mL/min. Three independent LC-MS/MS runs were performed for each sample.

The samples were ionized by positive ion electrospray in the LTQ-FTMS using 5 kV voltage on the needle, with a capillary voltage of 35 V, capillary temperature of 275 °C, and tube lens voltage of 110 V. The tandem mass spectra were obtained in the ion trap of the LTQ-FTMS with an isolation window of 1 amu and normalized collision energy of 35 eV, with an activation Q of 0.250 and activation time of 30 milliseconds. Detection was done in the ion trap of the LTQ-FTMS.

### 3.8. Statistical Analysis

Xcalibur software (Thermo Scientific) was used to integrate the area under the reconstructed selected ion chromatograms of the product ions. Xcalibur was also used to obtain the retention time and MS/MS spectrum of each analyte. IGOR Pro software (version 6.0.1.9, WaveMetrics, Lake Oswego, OR, USA) was used to analyze calibration curves and calculate organ concentrations. Organ concentrations are reported as mean ± standard deviation and are based on 4–7 independent extractions. Data were corrected to pmol/g wet tissue or pmol/10^6^ cells to normalize between organs and cells.

### 3.9. Method Validation Test

A frozen rat brain (1.22 g wet tissue) was homogenized in 4 mL pre-chilled acetonitrile/methanol/water buffer (1:2:2, v/v/v), as described above. The crude lysate was split into three equal portions (~1 mL each, samples 1–3) and transferred to individual tubes. The centrifuge tube was washed with 1.2 mL water; the wash was split equally into each tube (0.4 mL each). 100 pmol IS (8-Br-cAMP) was added to each sample. 60 pmol of 3',5'-cCMP was added to sample 2 following homogenization, while 60 pmol of 3',5'-cCMP was added to sample 3 after heating. LC-MS/MS quantitation was performed independently on each sample (refer to Experimental section 3.6 and Experimental section 3.7).

### 3.10. Method Reproducibility Test

A frozen rat brain (1.55 g wet tissue) was homogenized in 5 mL pre-chilled acetonitrile/methanol/water buffer, as described above (refer to heat extraction from rat organs section). The crude lysate was split into 3 equal portions (~2 mL each) and transferred to individual tubes. The centrifuge tube was washed with 1.2 mL water, which was split equally into each tube (0.4 mL each). Heat extraction and LC-MS/MS experiments were performed independently on each sample (refer to Experimental section 3.4, Experimental section 3.6 and Experimental section 3.7).

### 3.11. Synthesis of 2',3'-cIMP

2',3'-cIMP was synthesized by deamination of 2',3'-cAMP, as previously described [62,63,64]. A solution of NaNO_2_ (26 mg) in 100 μL water was added to 50 mg of 2',3'-cAMP (MP Biomedical) in glacial acetic acid (0.8 mL). The solution was stirred at room temperature for 6 hrs. Three equal portions of NaNO_2_ (60 mg total) were added over the 6 h period and the reaction was allowed to continue stirring for an additional 30 h (36 h total). After 36 h, the reaction was evaporated *in vacuo* to dryness. The product was purified by anion exchange chromatography using DEAE cellulose resin in carbonate form and eluted with a gradient of 0.005 M ammonium bicarbonate (pH 7.8) to 0.1 M ammonium bicarbonate (pH 7.0). The high resolution MS (calculated: 331.04515, observed: 331.04525), ^1^H, ^13^C, and ^31^P NMR, and UV spectra confirmed the 2',3'-cIMP structure.

## 4. Conclusions

In summary, we have described a sensitive, efficient and versatile method using standard instrumentation to extract and quantify cNMPs in mammalian tissues and cellular systems. The optimized LC-MS/MS protocol can separate and quantify all eight cNMPs studied. By utilizing the present method, we have established the tissue distributions of a panel of cNMPs, allowing for future studies on their cellular roles. Using commercially available 8-Br-cAMP greatly reduced the cost of experiments, relative to the use of isotopically labelled standards, thereby making the protocol economically feasible for a wide variety of researchers. Furthermore, this study allows for quantification of eight cNMPs simultaneously, which provides the opportunity to compare levels of multiple cNMPs in the same system and examine the relationship between tissue levels of cNMPs and organ function. Since the signalling networks for some cNMPs may be intertwined (e.g., 3',5'-cIMP is likely a deamination product of 3',5'-cAMP in cells) [7], correlation of cNMPs levels in various rat organs, and their alterations in disease states, will provide insights into additional potential signalling pathways.

## Supplementary Files

Supplementary File 1
