# Supplementary material for: A Facile and Sensitive Method for Quantification of Cyclic Nucleotide Monophosphates in Mammalian Organs: Basal Levels of Eight cNMPs and Identification of 2',3'-cIMP
Source: Biomolecules. 2014 Dec 12;4(4):1070–92. doi: 10.3390/biom4041070 (PMC4279170; doi:10.3390/biom4041070)
Supplement: Supplementary File 1 [file biomolecules-04-01070-s001.pdf]

Supplementary Information

Table S1. Comparison of ionization methods for LC-MS/MS analysis of cNMPs. Data for four cNMPs (ratios of peak areas of cNMP to internal standard) are listed. Positive ion mode exhibits improved sensitivity, particularly at lower cNMP concentrations.

	**A(2',3'-cAMP)/ A(IS81)**	**A(3',5'-cAMP)/ A(IS81)**	**A(2',3'-cGMP)/ A(IS81)**	**A(3',5'-cGMP)/ A(IS81)**
Positive ion: 2.5 μM	3.07	2.15	1.87	3.35
Negative ion: 2.5 μM	1.52	1.90	1.99	1.27
Positive ion: 1 μM	1.65	1.03	1.24	1.78
Negative ion: 1 μM	0.68	0.82	0.93	0.56
Positive ion: 0.5 μM	0.82	0.51	0.60	0.86
Negative ion: 0.5 μM	0.35	0.43	0.48	0.27

Table S2. Thermal stability test (μM). Samples dissolved in water were treated at 60 °C for 10 min.

	**2',3'-cAMP**	**3',5'-cAMP**	**2',3'-cCMP**	**3',5'-cCMP**	**2',3'-cGMP**	**3',5'-cGMP**	**3',5'-cIMP**	**8-Br-cAMP**
**Sample conc.**	46.4	50.3	48.6	60.8	40.5	54.7	30.3	45.8
**Conc. after treating with heat**	59.4	48.1	69	70.6	35.2	62.7	42.1	50.4
**% to reference sample**	128%	95.6%	141%	116%	87%	114%	138%	110%

Table S3. Deaminase activity test (pmol/g wet tissue), concentrations are reported as mean ± range (each sample was analyzed twice on LC-MS/MS). Liver A and B were from one liver. 40 pmol of 3',5'-cAMP was added to liver B.

	**3',5'-cAMP**	**2',3'-cIMP**	**3',5'-cIMP**
**Liver A**	3.90 ± 0.82	1.85 ± 0.19	1.76 ± 0.02
**Liver B**	1844.14 ± 106.63	2.34 ± 0.17	2.28 ± 0.40

Table S4. Comparison of extraction efficiency of the internal standard (IS; 8-Br-cAMP) at different addition times. A rat brain was homogenized, split into two equal parts, and IS was added either to crude lysate (Figure 2, step 2) or following centrifugation (Figure 2, step 4). 3',5'-cAMP was quantified to provide a reference. No significant difference in peak areas was detected.

**Sample**	**Internal Standard**	**3',5'-cAMP**
IS addition to crude lysate	123,458 ± 6715	272,637 ± 12635
IS addition post-heating	140,722 ± 7070	267,800 ± 13043

Table S5. Intra-run precision and accuracy data in tabulated form. Extractions and measurements were performed in rat brains (see Figure 5).

	**Measured (pmol)**	**Precision (RSD in %)**	**Accuracy (%)**
3',5'-cCMP (60 pmol)	63.3 ± 7.4	11.7%	105.4%
3',5'-cCMP (60 pmol)	70.8 ± 16.6	23.5%	117.9%

Table S6. Measured concentrations of 8 cNMPs in various rat organs reported as mean ± SD (pmol/g wet tissue). Each cNMP was calculated using 4–7 individual organs as replicates, and each sample was analyzed in 2–3 separate runs.

	2',3'-cAMP	3',5'-cAMP	2',3'-cCMP	3',5'-cCMP	2',3'-cGMP	3',5'-cGMP	2',3'-cIMP	3',5'-cIMP
				**Brain**				
Rat 1	8.73	497.03 ± 243.75	N/D	N/D	N/D	2.71	0.90 ± 0.52	N/D
Rat 2	N/D	162.61 ± 31.21	2.50*	N/D	6.48 ± 1.19	5.22 ± 1.79	N/D	N/D
Rat 3	11.50	351.00 ± 24.84	2.63	0.67 ± 0.09	17.49 ± 11.86	22.09 ± 4.82	N/D	N/D
Rat 4	N/D	458.28 ± 67.80	N/D	3.75 ± 0.01	22.09 ± 0.63	11.12 ± 4.05	0.17 ± 0.37	N/D
Ave brain	10.11 ± 1.96	367.23 ± 149.75	2.57 ± 0.07	2.21 ± 1.54	15.35 ± 8.02	10.29 ± 8.62	0.54 ± 0.37	N/D
				**Heart**				
	**2',3'-cAMP**	**3',5'-cAMP**	**2',3'-cCMP**	**3',5'-cCMP**	**2',3'-cGMP**	**3',5'-cGMP**	**2',3'-cIMP**	**3',5'-cIMP**
Rat 1	N/D	849.05 ± 10.44	8.73	N/D	4.17	N/D	N/D	N/D
Rat 2	N/D	442.14 ± 46.57	4.57 ± 0.27	3.60 ± 0.04	13.63 ± 6.16	7.61 ± 3.04	BQL	N/D
Rat 3	N/D	365.55 ±43.38	4.22	3.62 ±0.10	7.27 ±4.04	6.58 ±1.98	N/D	N/D
Rat 4	28.35	887.34 ± 156.06	N/D	10.96 ±0.75	28.06 ± 7.03	15.98 ± 12.12	BQL	N/D
Rat 5	7.30	439.40 ± 69.43	N/D	9.49 ± 1.12	20.90 ± 4.48	11.16 ±4.60	0.07 ± 0.03	2.86
Female rat 1	32.84 ± 29.36	443.07 ± 62.82	N/D	3.76 ± 4.06	5.63 ± 0.03	N/D	N/D	N/D
Ave heart	22.83 ± 13.63	571.09 ±232.33	5.84 ±2.51	6.29 ± 3.63	13.30 ± 9.52	10.33 ± 4.25	0.07	2.86
				**Lung**				
Rat 1	15.67 ± 20.89	515.52 ± 48.28	291.44 ± 425.18	N/D	6.67 ± 3.74	5.98	N/D	N/D
Rat 2	N/D	212.66 ± 4.98		18.89 ± 0.81	4.74 ± 1.42	3.28 ± 0.61	N/D	N/D
Rat 3	N/D	914.96 ± 130.83	7.35 ± 1.47	9.60 ± 0.97	25.42 ± 1.46	25.49 ± 12.15	N/D	N/D
Rat 6	19.58 ± 2.42	554.21 ± 50.42	68.73 ± 6.94	0.78	N/D	12.24	N/D	N/D
Rat 7	17.42	227.84 ± 114.58	N/D	1.45	N/D	N/D	N/D	N/D
Female rat 2	29.21 ± 2.97	607.29 ± 106.08	2.04	1.69 ± 1.00	27.73 ± 2.64	N/D	N/D	N/D
Ave lung	20.48± 6.04	505.58 ± 262.05	92.39 ± 136.11	6.48 ± 7.81	16.14± 12.11	11.75 ± 9.90	N/D	N/D
				**Kidney**				
Rat 1	20.31 ± 11.65	417.72 ± 13.15	187.15 ± 24.26	N/D	12.66	N/D	N/D	3.05 ± 1.83
Rat 2	28.60 ± 11.02	646.05 ± 143.88	14.80 ± 11.99	36.68 ± 6.37	11.61	N/D	BQL	N/D
Rat 3	21.88 ± 12.04	647.25 ± 372.78	6.58 ± 0.66	7.45	N/D	N/D	N/D	N/D
Rat 4	17.96 ± 7.53	452.31 ± 190.19	3.94 ± 1.97	12.06	2.38	0.02	BQL	4.16
Rat 6	46.08 ± 11.72	305.35 ± 59.38	N/D	N/D	3.81 ± 1.22	N/D	N/D	4.16
Rat 7	56.20 ± 4.84	513.95 ± 63.81	438.16 ± 262.27	N/D	10.99 ± 0.19	N/D	N/D	N/D
Female rat 2	72.68 ± 48.38	445.49 ± 43.87	10.88 ± 5.03	1.22	9.64 ± 2.00	8.50 ± 6.14	N/D	N/D
Ave kidney	37.67 ± 21.06	489.73 ± 124.08	110.25 ± 175.77	14.35 ± 15.53	8.51 ± 4.33	4.26 ± 6.00	BQL-N/D	3.79 ± 0.64

Table S6. *Cont.*

	2',3'-cAMP	3',5'-cAMP	2',3'-cCMP	3',5'-cCMP	2',3'-cGMP	3',5'-cGMP	2',3'-cIMP	3',5'-cIMP
				**Spleen**				
	**2',3'-cAMP**	**3',5'-cAMP**	**2',3'-cCMP**	**3',5'-cCMP**	**2',3'-cGMP**	**3',5'-cGMP**	**2',3'-cIMP**	**3',5'-cIMP**
Rat 1	21.80 ± 3.47	724.48 ± 14.16	114.56 ± 79.19	N/D	11.84	N/D	N/D	3.39
Rat 2	30.07 ± 15.39	339.83 ± 7.73	9.33 ± 2.07	5.74 ± 0.10	17.07 ± 5.51	6.47 ± 1.24	N/D	N/D
Rat 3	79.60 ± 32.62	1050.12 ± 290.46	191.18 ± 91.08	20.14 ± 4.66	47.20 ± 7.28	25.50 ± 5.48	BQL	N/D
Rat 4	11.52 ± 2.86	694.34 ± 84.05	N/D	7.43 ± 0.37	26.54 ± 3.39	17.98 ± 2.01	0.05 ± 0.01	N/D
Female rat 1	214.65 ± 109.03	604.31 ± 249.78	123.48 ± 117.42	14.81	N/D	N/D	N/D	N/D
Ave spleen	71.53 ± 84.17	682.62 ± 255.24	109.63 ± 75.11	11.10 ± 7.87	25.66 ± 15.59	16.65 ± 9.59	0.05	3.39
				**Liver**				
Rat 8	N/D	144.31	15.62	N/D	N/D	N/D	1.7	N/D
Rat 9	N/D	195.03	64.70	N/D	N/D	N/D	N/D	1.25
Rat 10	12.63	617 ± 192.93	6.10 ± 1.54	46.96 ± 7.08	7.07 ± 1.39	N/D	2.5	7.95
Rat 11	6.23	84.97 ± 23.22	1.19 ± 0.17	2.48	N/D	N/D	N/D	2.39
Ave liver	9.43 ± 3.2	260.46 ± 242.26	21.90 ± 29.16	24.72 ± 22.24	7.07 ± 1.40	N/D	2.1 ± 0.4	3.86 ± 3.59

* Only one injection was evaluated for numbers without standard deviation; N/D—Not detected, concentration below LOD; BQL—below quantification limit; detected, but level too low to quantify.

Figure S1. FT-MS of 3',5'-cAMP (**A**) and 2',3'-cAMP (**B**). While FT-MR analysis of cNMPs results in excellent sensitivity, the regioisomers yield identical mass spectra and cannot be distinguished.

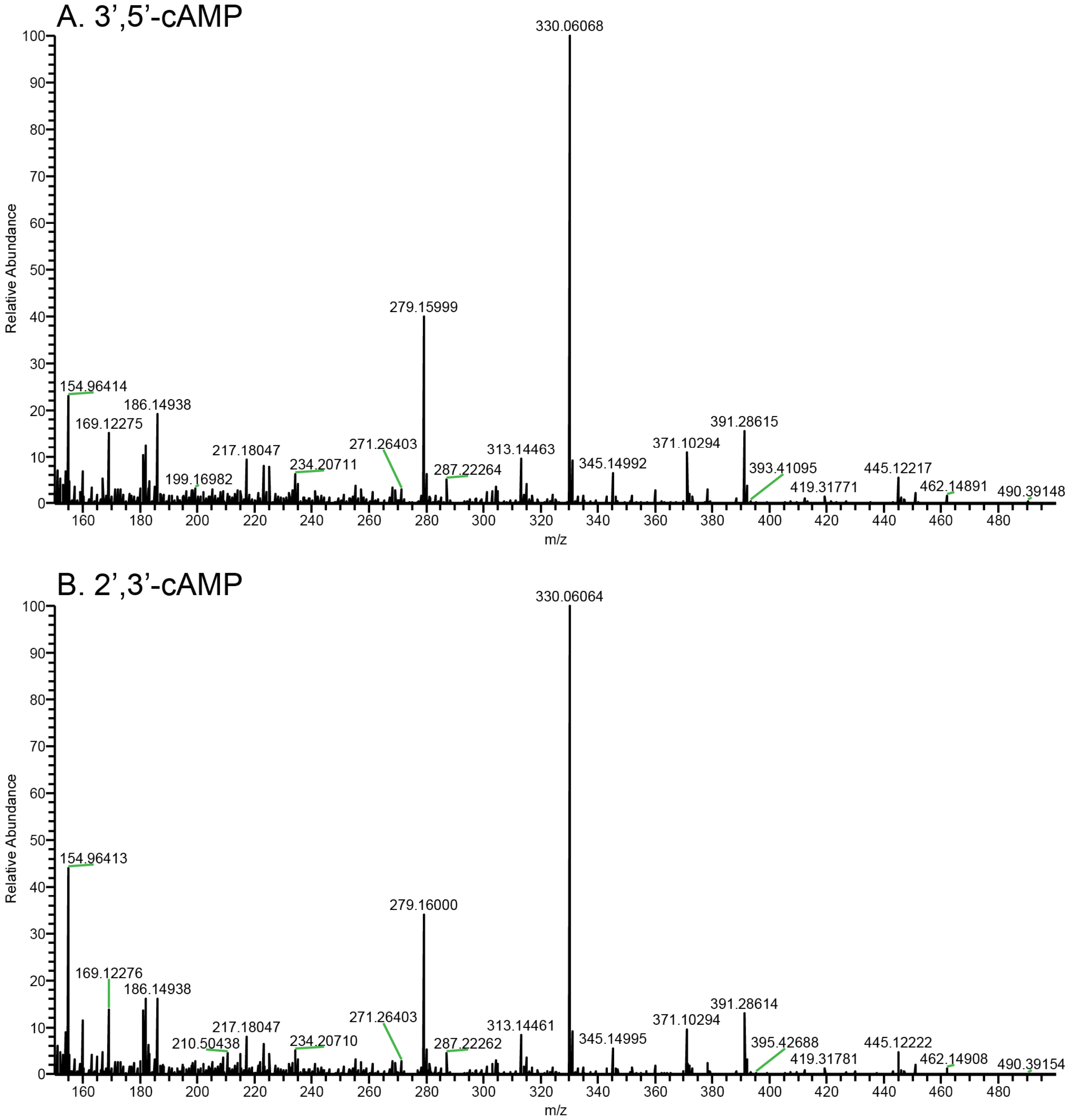

Figure S2. MS/MS spectrum of authentic 2',3'-cGMP.

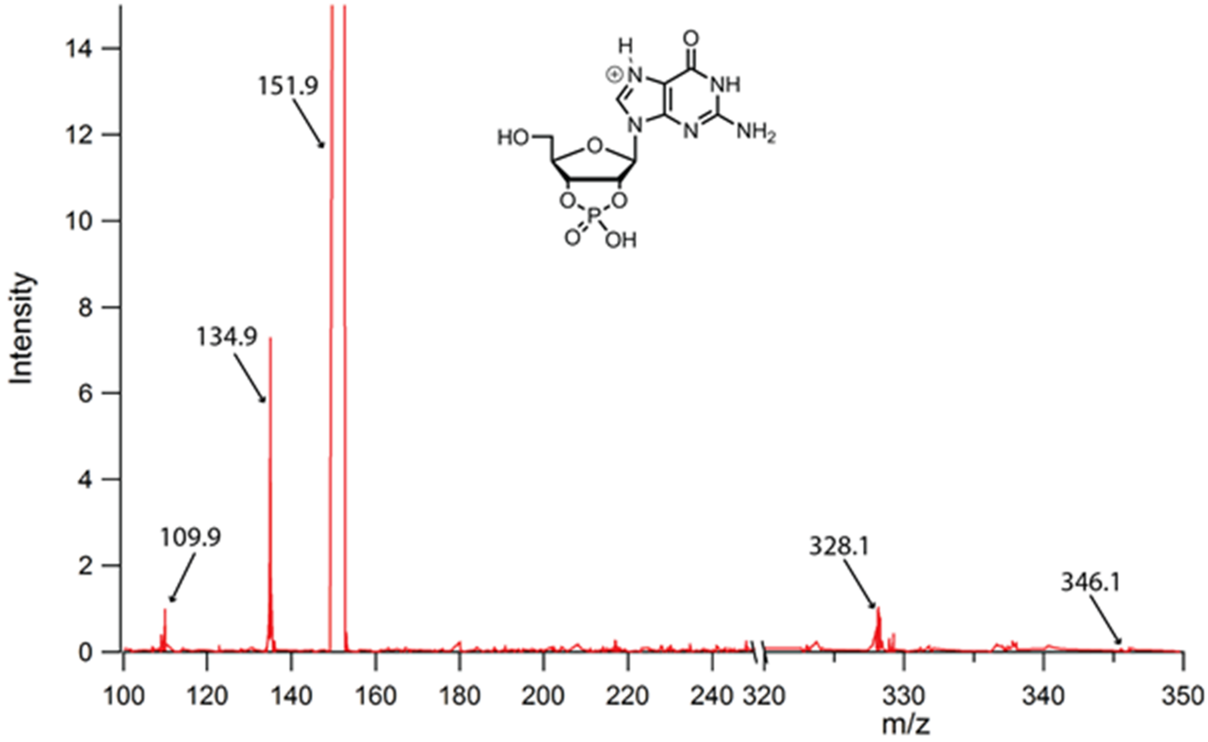

Figure S3. MS/MS spectrum of extracted 2',3'-cGMP in rat heart.

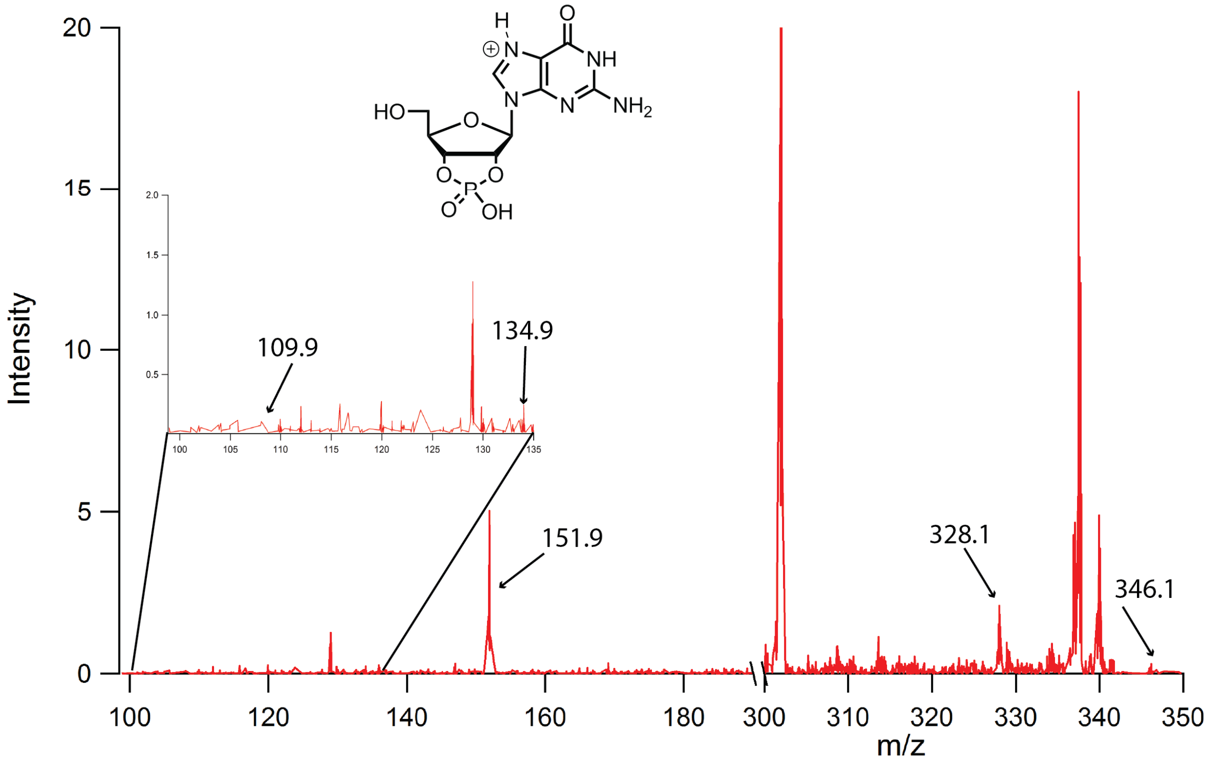

Figure S4. High resolution MS (**A**) and MS/MS (**B**) spectra of authentic 2',3'-cIMP. 2',3'-cIMP calculated: 331.04515; observed: 331.04525. $[BH_2]^+$ calculated: 137.04635; observed: 136.91667.

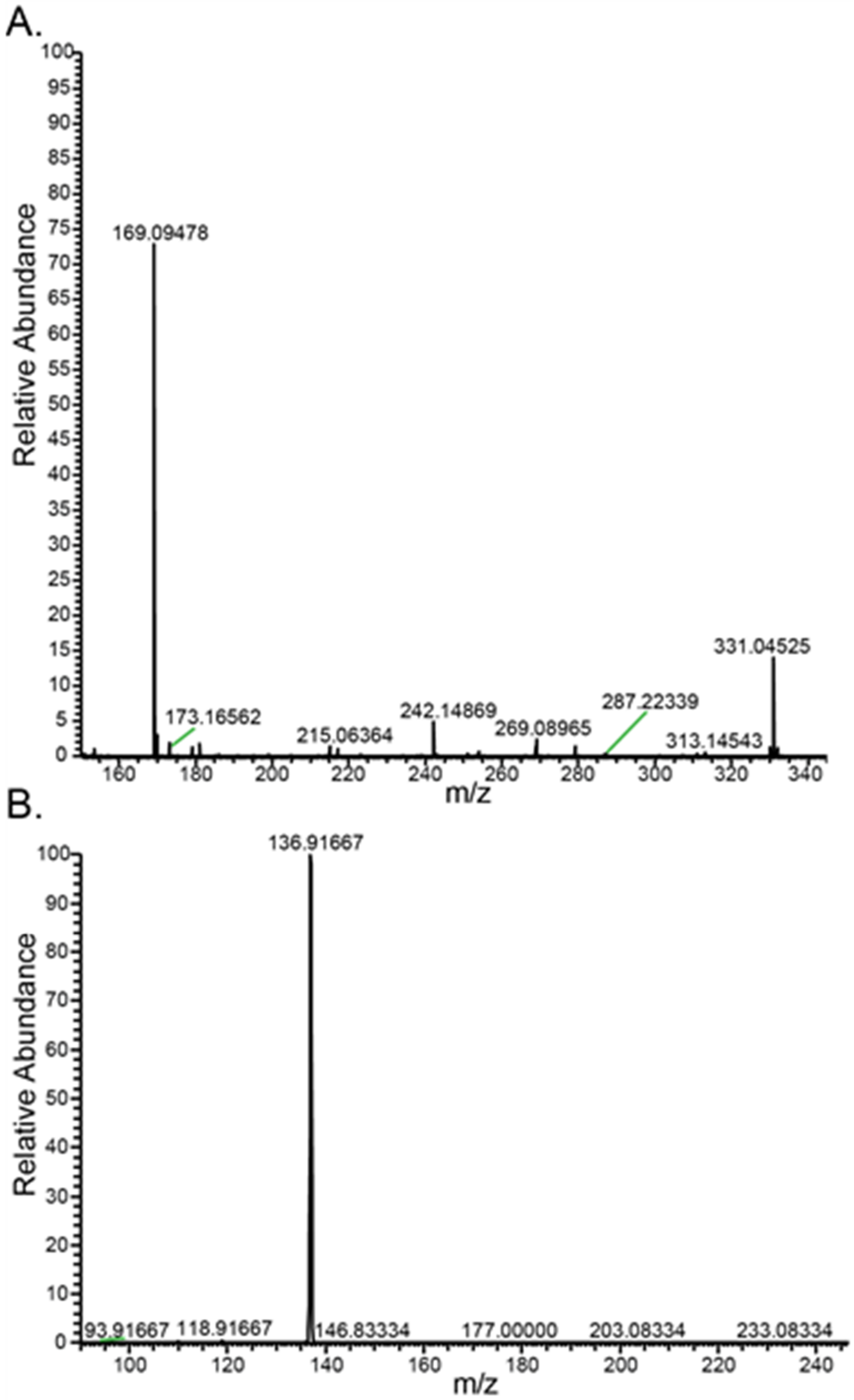

© 2014 by the authors; licensee MDPI, Basel, Switzerland. This article is an open access article distributed under the terms and conditions of the Creative Commons Attribution license (http://creativecommons.org/licenses/by/4.0/).
